# Characteristics and outcomes of patients with culture negative septic shock compared with patients with culture positive septic shock: a retrospective cohort study

**DOI:** 10.1186/cc12908

**Published:** 2013-11-05

**Authors:** Shravan Kethireddy, Amanda Bengier, H Lester Kirchner, R Bruce Light, Yazdan Mirzanejad, Dennis Maki, Yaseen Arabi, Steven Lapinsky, David Simon, Aseem Kumar, Joseph E Parrillo, Anand Kumar

**Affiliations:** 1 Section of Critical Care Medicine and Infectious Diseases, Geisinger Medical Center, Danville, PA, USA; 2 Clinical Innovation and Biostatistics, Division of Internal Medicine, Geisinger Medical Center, Danville, PA, USA; 3 Section of Critical Care, Section of Infectious Diseases, University of Manitoba, Winnipeg, MB, Canada; 4 Surrey Memorial Hospital, Surrey, BC, Canada; 5 University of Wisconsin Hospital and Clinics, Madison, WI, USA; 6 King Saud Bin Abdulaziz University for Health Sciences, Riyadh, Saudi Arabia; 7 Section of Critical Care Medicine, University of Toronto, ON, Canada; 8 Rush University, Chicago, IL, USA; 9 Laurentian University, Sudbury, ON, Canada; 10 Hackensack University Medical Center, Hackensack, NJ, USA

## Background

Previous studies have identified that nearly 30% of patients with severe sepsis and septic shock lack a definitive microbial etiology. The characteristics and outcomes of culture negative septic shock are not well defined despite large epidemiologic studies on septic shock

## Materials and methods

Retrospective nested cohort study of 2,651 patients with culture-negative septic shock and 6,019 culture-positive septic shock patients derived from a trinational, 8,760-patient database of patients with septic shock between 1989 and 2008.

## Results

In total, 30.6% of cases of septic shock cases were identified as culture-negative within the database. Patients with culture-negative septic shock (CNSS) experienced similar ICU mortality as did those with culture-positive septic shock (CPSS) (41.7% vs. 40.5% *P *= 0.276) and identical overall hospital mortality (51.9% vs. 51.9% *P *= 0.976). Severity of illness was similar between CNSS and CPSS (median APACHE II 25 (IQR 6 to 54) vs. 25 (IQR 4 to 70) respectively). Initial and 6-hour lactate levels were also similar among CNSS and CPSS patients (mean 4.4 vs. 4.1, *P *= 0.237 and mean 4.0 vs. 4.1, *P *= 0.221 respectively). Interestingly CNSS patients were significantly more likely to be hypothermic than CPSS patients (temperature <36°C 18.9% vs. 15.3%, *P *< 0.0001). CNSS patients presented significantly more often from the community (63.3% vs. 58.0%, *P *< 0.0001), where patients with CPSS presented more often with nosocomial infections (36.7% vs. 42.0%, *P *< 0.0001). Gastrointestinal and respiratory tract infections were the predominant sources of infection in both groups. However, CNSS patients with respiratory tract infections experienced lower mortality than their CPSS counterparts (49.6% vs. 56.3%, *P *= 0.008) but similar mortality rates with gastrointestinal infections (61.0% vs. 58.2%, *P *= 0.289) (Tables 1 and 2).

**Table 1:** Comparison of variables of culture-positive and culture-negative septic shock

Variable			
Sex			0.3725
Female	2,565 (42.6)	1,157 (43.6)	
Male	3,454 (57.4)	1,494 (56.4)	
Age			<0.0001
Mean	62.0 (16.1)	63.9 (16.3)	
Median	64.0	67.0	
Range	16 to 102	16 to 101	
Admit source			0.0567
Surgery	86 (3.2)	53 (4.3)	
ER	2,245 (84.1)	990 (80.5)	
Medical	334 (12.5)	180 (14.7)	
TxER	3 (0.1)	3 (0.2)	
TxICU	2 (0.1)	2 (0.2)	
TxWard	0 (0)	1 (0.1)	
Survival			
15	3,785 (62.9)	1,598 (60.3)	0.0213
30	3,275 (54.4)	1,399 (52.8)	0.1585
90	2,906 (48.3)	1,283 (48.4)	0.9204
ICU	3,583 (59.5)	1,545 (58.3)	0.2760
Overall	2,895 (48.1)	1,276 (48.1)	0.9760
ICU LOS			<0.0001
Missing (%)	0	0	
Mean	11.0 (13.4)	9.8 (13.7)	
Median	7.0	6.0	
Range	1.0 to 215.0	1.0 to 314.0	
Hospital LOS			<0.0001
Missing (%)	0	0	
Mean	26.3 (34.1)	23.1 (31.1)	
Median	15.0	12.2	
Range	0.5 to 370.0	0.3 to 314.0	
APACHE			0.4450
Missing (%)	375 (6.2)	179 (6.8)	
Mean	25.7 (8.1)	25.7 (8.3)	
Median	25.0	25.0	
Range	4 to 70	6 to 54	
Days to extubation			0.2414
Mean	6.5 (9.5)	6.2 (9.2)	
Median	4.0	3.0	
Range	0.0 to 117.0	0.0 to 100.0	
Days on pressors			0.1927
Missing (%)	1,878	892	
Mean	3.6 (3.7)	3.3 (3.2)	
Median	3.0	2.0	
Range	0.0 to 54.0	0.0 to 34.0	
Temperature			<0.0001
Mean	37.6 (1.7)	37.4 (1.7)	
Median	38.0	36.2	
Range	27.3 to 42.7	30.4 to 42.7	
<36°C	869 (15.3)	473 (18.9)	<0.0001
>38°C	2,707 (47.7)	1,075 (42.9)	<0.0001
>38.3°C	2,264 (39.9)	878 (35.0)	<0.0001
Infection source			<0.0001
Community	3,491 (58.0)	1,677 (63.3)	
Nosocomial	2,528 (42.0)	974 (36.7)	
Lactate - baseline			0.2377
Missing (%)	5,328 (88.5)	2,328 (87.8)	
Mean	4.3 (3.8)	4.4 (4.5)	
Median	3.1	2.8	
Range	0.3 to 26.4	0.3 to 26.7	
Lactate - 6 hours			0.2214
Missing (%)	5,132 (85.3)	2,204 (83.1)	
Mean	4.1 (3.7)	4.0 (3.8)	
Median	2.8	2.6	
Range	0.1 to 25.8	0.4 to 23.1	
Lactate - 24 hours			0.4919
Missing (%)	5,164 (85.8)	2,236 (84.3)	
Mean	3.7 (4.1)	4.1 (5.0)	
Median	2.4	2.3	
Range	0.3 to 54.4	0.2 to 37.6	

**Table 2:** Comparison of major sites of infection

	Culture-positive	Culture-negative	*P *value
**Respiratory infection**			
*n *(%)	2,172 (63.9)	1,228 (36.1)	
ICU LOS (median, IQR)	8.0 (4, 16)	6.7 (3, 12.8)	<.0001
Hospital LOS (median, IQR)	16.0 (6, 32)	13.0 (5, 26)	0.0024
15-day survival (*n*, %)	1,344 (61.9)	782 (63.7)	0.4761
Hospital survival (*n*, %)	949 (43.7)	619 (50.4)	0.0086
APACHE (mean, SD)	26.4 (8.0)	25.4 (8.0)	<.0001
**Gastrointestinal infection**			
*n *(%)	1,476 (58.9)	1,030 (41.1)	
ICU LOS (median, IQR)	6.5 (3, 13)	5.0 (3, 11)	0.0153
Hospital LOS (median, IQR)	15 (5.7, 33)	11.1 (3, 29)	0.0059
15-day survival (*n*, %)	877 (59.4)	542 (52.6)	0.0002
Hospital survival (*n*, %)	617 (41.8)	402 (39.0)	0.2892
APACHE (mean, SD)	25.5 (8.2)	26.1 (8.5)	0.1180

**Figure 1:**
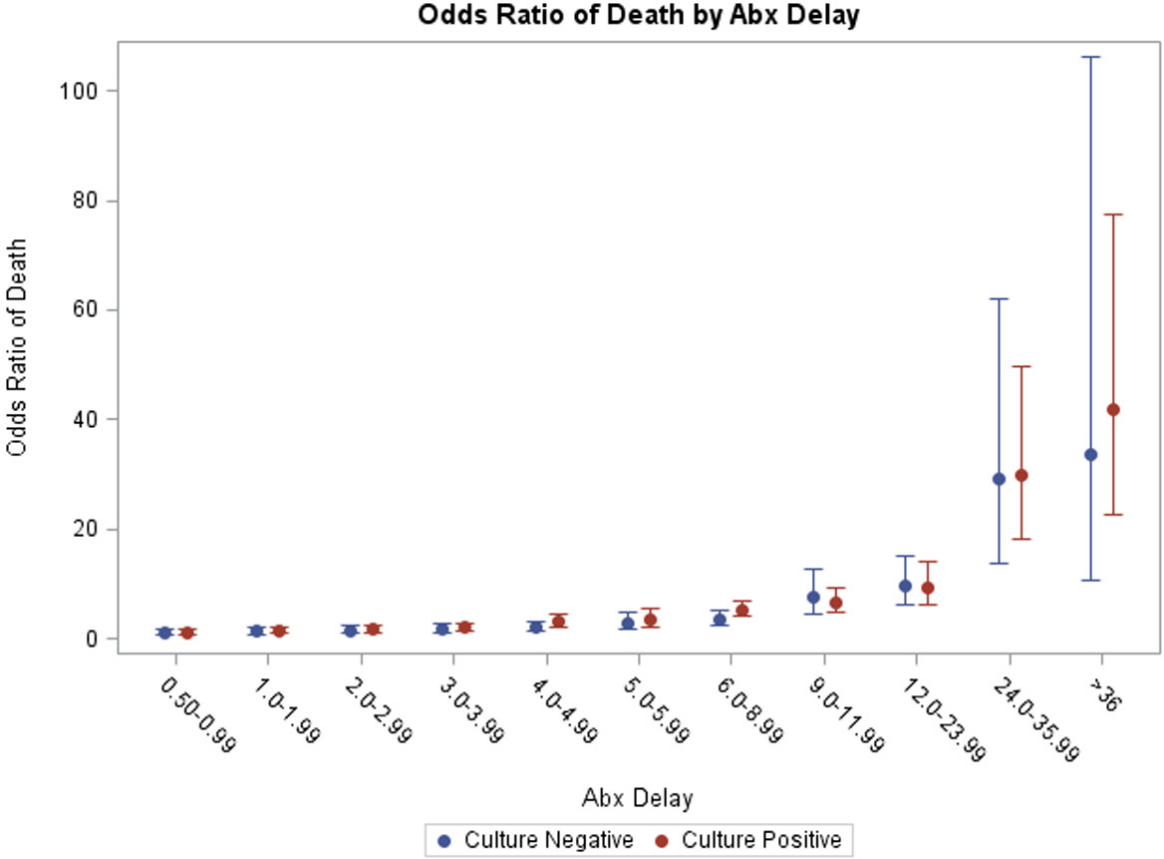
**Odds ratio of death by antibiotic delay in culture-positive and culture-negative septic shock**.

Similar to our previous findings, we identified by the second hour after onset of persistent/recurrent hypotension that the in-hospital mortality rate was significantly increased relative to receiving therapy within the first hour (odds ratio, 1.62; 95% CI, 1.21 to 2.15; *P *< 0.001) in the CPSS group. Following increasing delays in the administration of appropriate antimicrobial therapy over the first 6 hours after the onset of hypotension, patients in both groups experienced nearly congruent, significant increases in hospital mortality; at 6 hours the CNSS group (odds ratio, 2.87; 95% CI, 1.72 to 4.78; *P *< 0.0001) and the CPSS group (odds ratio, 3.44; 95% CI, 2.17 to 5.48; *P *< 0.0001) (Figure 1). Survival differences between these time intervals are not significantly different in patients with CNSS and CPSS.

## Conclusions

Patients with CNSS behave similarly to CPSS patients in nearly all respects. As with bacterial septic shock, early appropriate antimicrobial therapy appears to improve mortality. Earlier recognition of infection is the most obvious effective strategy to improve hospital survival. Optimal duration of therapy is not well defined among patients with CNSS. In addition to early, appropriate antimicrobial therapy, use of de-escalation strategies such as serial procalcitonin levels may be useful to determine the length of empiric broad-spectrum antimicrobial use in this population.

